# Behind the scenes of a 7T MRI clinical study in Alzheimer's disease: challenges and recommendations for future research

**DOI:** 10.3389/fnagi.2026.1731961

**Published:** 2026-06-19

**Authors:** Maria Dobrushina, Friedrich Krohn, Sayyeda Chandni, Linda Dietrich, Wenzel Glanz, Michaela Butryn, Dorothea Hämmerer, Lucía Penalba-Sánchez

**Affiliations:** 1 Institute of Cognitive Neurology and Dementia Research (IKND), Otto-von-Guericke University Magdeburg, Magdeburg, Germany; 2 German Center for Neurodegenerative Diseases (DZNE), Magdeburg, Germany; 3 University Hospital of Child and Adolescent Psychiatry and Psychotherapy, University of Bern, Bern, Switzerland; 4 Department of Neurology, Otto-von-Guericke University, Magdeburg, Germany; 5 Institute of Cognitive Neuroscience, University College London (UCL), London, United Kingdom; 6 Center for Behavioral Brain Sciences (CBBS), Magdeburg, Germany; 7 Department of Psychology, University of Innsbruck, Innsbruck, Austria

**Keywords:** Alzheimer's disease, clinical studies, eligibility, MRI, recruitment, 7 Tesla MRI

## Abstract

Combining pharmacological interventions with neuroimaging in Alzheimer's disease (AD) research presents logistical and methodological challengesparticularly during early study phases, such as recruitment and study visits. Yet, these challenges are rarely reported in detail. This perspective article shares early-phase experiences from a 7-tesla (7T) fMRI clinical study involving individuals with mild cognitive impairment due to AD (MCI-AD) and mild Alzheimer's disease dementia (mild ADD), highlighting recruitment hurdles and offering practical recommendations. From a pool of 1,001 patients, 476 had a clinical diagnosis of MCI-AD or mild ADD; after pre-selection and screening, only 48 participants (10%) met all inclusion criteria. Major exclusion factors included metal or tattoos (8%), missing surgical documentation (2%), beta-blocker use (27.5%), recent cancer/chemotherapy or brain radiation (1%), and lack of interest (20%). Effective communication, often requiring caregiver assistance, was essential for obtaining accurate medical histories and improving adherence. Internally, clear team coordination supported scheduling and protocol compliance. While strict eligibility criteria improve data quality, they can substantially limit recruitment and feasibility in pharmacological and high-field MRI drug studies. We propose strategies to optimize recruitment and screening, facilitate data collection, and balance scientific rigor with real-world clinical feasibility.

## 1 Introduction

Alzheimer's disease (AD) is the most common cause of dementia, affecting about 32 million people worldwide (Gustavsson et al., 2023) andit is expected to double every 20 years (Prince, 2015). While progress has been made in harmonizing study protocols (Ellis et al., 2009), operational factors like recruitment and exclusion criteria are often under-reported. Furthermore, the incidence of MCI-AD and ADD remains around 0.1% and 1% in the German population (Bohlken et al., 2021). Including only participants who present MCI-AD or mild ADD and are MRI and drug compatible, complicates recruitment, especially when time and funding are limited time. This raises the question of whether to relax inclusion criteria for larger samples, despite increased heterogeneity. Drawing on our experience from a 7T pharmacological MRI study in well-characterized individuals with MCI-AD and ADD (see Supplementary material for diagnostic details), we provide practical recommendations across all study stages, from participant identification to testing, and highlight which criteria may be adapted and which should remain stringent.

## 2 Scientific context: a 7T AD atomoxetine study

The Locus Coeruleus (LC) is a small structure in the brainstem and the primary source of central noradrenaline. It is involved in modulating sleep, arousal, memory encoding and consolidation (Dahl et al., 2023) and attentional control (Dahl et al., 2022). The LC is one of the first regions to be affected by Alzheimer's disease (AD) (Braak et al., 2011; Harley et al., 2021; Liebe et al., 2022; Penalba-Sánchez et al., 2024), possibly as early as in a pre-clinical stage (Liebe et al., 2022; Penalba-Sánchez et al., 2024). Atomoxetine, a noradrenergic reuptake inhibitor, has been linked to increased noradrenergic plasma and reduced CSF and pTau181 levels in MCI-AD (Levey et al., 2022) and might thus also improve brain and cognitive function during early stages of AD.

To investigate LC-noradrenergic function individuals with MCI-AD and mild ADD visited the clinic four times and completed a stop signal task (SST), an incidental memory encoding task (IMT) during an fMRI scan and a recognition memory task outside the scan.During the SST (visits 1 and 3), participants responded to, inhibited, or withheld responses to visual stimuli (see Supplementary material for further details). During the IMT, participants viewed 80 emotional and neutral scenes (incidental encoding) inside the scanner, followed by a recognition memory task the next day. This procedure was repeated 1 week later under the alternate condition (atomoxetine or placebo). Non-MRI eligible participants completed the encoding phase outside the scanner.

## 3 Recruitment process

**Figure 1:**
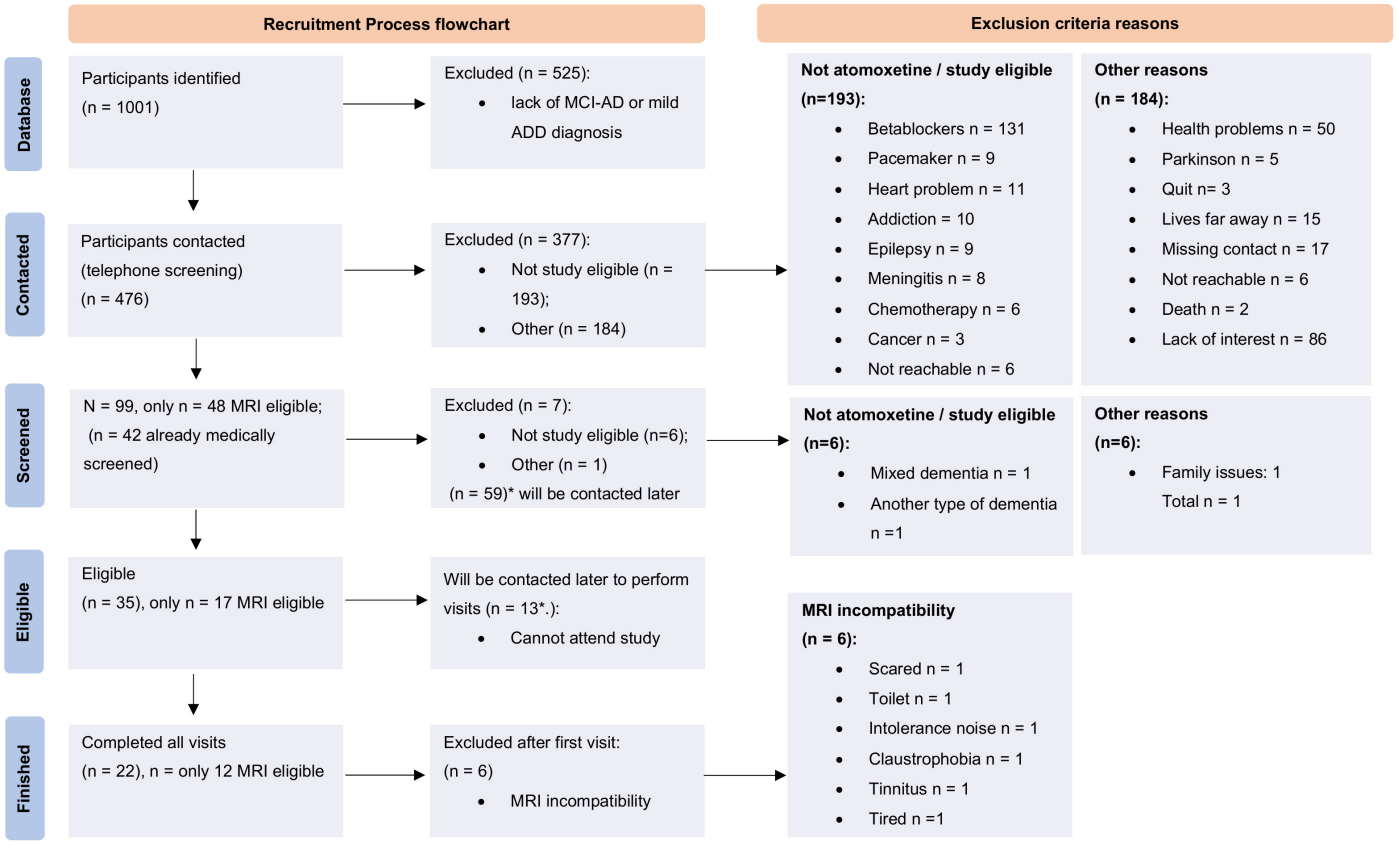
Shows the recruitment pipeline, exclusion criteria and the corresponding sample size. ^*^All participants included in the study will be/have already been conducting the experimental tasks with a dose of atomoxetine but only a subpart will also be undergoing MRI scans. As this is an ongoing study, fifty-nine participants will potentially attend the medical screening and thirteen participants who already passed the screening will be contacted again to schedule study appointments. However, many of these participants might be potential excluded, e.g., difficulties attending, caregiver not accompanying them, not interested anymore, participating in other studies, present a disease, develop moderated dementia, or other cause of exclusion. Considering our past statistics and the number of months until study deadline, we expect around a total of fifteen additional participants.

Participants were recruited from a university memory clinic database of 1001 records, from which 476 individuals with a confirmed MCI-AD or mild ADD diagnosis were identified as potential candidates. Following telephone screening, 377 were excluded due to medical contraindications to atomoxetine (e.g., betablockers use, cardiac conditions, epilepsy) or other reasons (e.g., lack of interest, not reachable), leaving 99 participants who met initial eligibility criteria. Of these, 42 were scheduled for medical screening based on availability, the remaining 59 are pending contact, as recruitment is ongoing. Medical screening confirmed eligibility in 35 participants, of whom 17 met the additional MRI compatibility criteria required for the 7T fMRI protocol. Six participants were subsequently excluded after the first study visit due to MRI incompatibility (e.g., claustrophobia, noise intolerance). In total, 22 participants successfully completed all four study visits, with 12 contributing 7T fMRI data. A detailed overview of exclusion criteria at each stage is presented in Figure 1.

### 3.1 Telephone screening

The 476 patients diagnosed with MCI-AD or mild ADD were contacted via telephone screening. During screening, individuals were assessed for MRI and atomoxetine eligibility.

Some participants were MRI ineligible due to metal implants or tattoos (*n* = 51, 10.7%) and ineligible for atomoxetine (*n* = 193, 40.5%), primarily due to beta-blocker use (*n* = 131, 27.5%) which might interfere by blocking beta-adrenergic receptors and reversing or canceling atomoxetine effects (Cismaru and Lupu, 2021). Additional reasons for exclusion were other health-related factors (*n* = 55, 11.5%), inability to reach them or residence far away from the clinic (*n* = 38, 7.9%), death (*n* =2, 0.4%) or lack of interest (*n* = 86, 18%).

To increase sample size, participants non-MRI eligible were also included in the study but they performed the tasks without MRI. Ultimately, *n* = 99, 20.7% were selected for medical screening and n = 42 were invited, depending on the availability of medical screening appointments.

### 3.2 Medical screening

During medical screening, participants' status was reconfirmed by reviewing their medical history, including clinical diagnosis and AD markers such as CSF beta amyloid and structural MRI. The screening required coordination among the study physician, patient, a caregiver, and researcher. As MRI slots As MRI slots must be reserved and paid for in advance, researchers should accurately as possible to avoid losing resources. To ensure safety, a blood panel, including a complete blood count and relevant markers, was conducted. Additionally, the Montreal Cognitive Assessment (MoCA) was administered to confirm that cognitive status matched that of previous visits (Nasreddine et al., 2005). In summary, after excluding 377 individuals during telephone screening from 476 pre-selected, 40 were invited for medical screening. *N* = 35 out of 40 met all criteria (i.e., for atomoxetine or MRI and atomoxetine) and were eligible for participation in the experimental phase, highlighting the importance and relative efficiency of the initial telephone screening.

### 3.3 Recommendations for recruitment

Nearly a third of participants (27.5%) were excluded due to beta-blocker use, highlighting how a single inclusion criterion can substantially limit sample size. Studies not requiring atomoxetine may therefore achieve considerably larger samples. We recommend thoroughly evaluating the impact of inclusion criteria on sample size before study onset. During telephone screening, asking specific rather than broad questions is particularly important given participants' memory impairment, for example, asking “Has there been any surgery on the hip?” rather than “Did you undergo any surgery in the past years?” reduces the risk of incidental exclusions. Further communication strategies are discussed in Section 5 and in the Discussion.

## 4 Experimental study visits

All participants passing the screening attended four experimental visits where behavioral and/or neuroimaging data for the study were acquired. While screening ensured safety and cognitive ability, in some cases, technical issues occurred. During SST and IMT fMRI, some struggled due to scanner discomfort (*n* = 2), fatigue (*n* = 1), or other reasons including restroom breaks (*n* = 3). Two participants presented difficulties reading the instructions when displayed inside the scanner. We recommend writing clear instructions and using intuitive tasks suitable for individuals with memory impairment. Stress also affected some and as such we recommend reassuring participants that perfection is not required in order, to reduce anxiety. In visits 2 and 4, during the memory test, where participants had to recognize the image scenes seen in visit 1 and 3, some with advanced MCI-AD and mild ADD failed to respond due to slow reaction times, but not necessarily due to memory. In this case, researchers assisted them in pressing the corresponding arrow keys. The pharmacodynamics of atomoxetine required a strict time-dependent visit protocol, enabling exploration of drug effects on behavior and fMRI activations. Verbose or disoriented participants caused delays. In these cases, we recommend redirecting the participants to the task in an empathic and assertive way.

## 5 The importance of communication

Clear communication is a cornerstone of any research study, but its importance is amplified when participants have memory or cognitive difficulties. Clear communication was essential for cooperation with participants, caregivers, and colleagues throughout all study stages. Caregiver involvement was particularly valuable during telephone and medical screening, where caregivers assisted in retrieving medical histories and collecting surgical documentation, information that participants with MCI-AD or mild ADD often could not recall accurately on their own.

We recommend keeping verbal instructions short and simple, as long sentences increased the risk of participants losing track. Written materials should complement oral explanations, and key information should always be communicated when the caregiver is present.

The tone and attitude of researchers and staff also played a crucial role, a confident, friendly, and calm approach improved participant comfort and performance. Clear pronunciation was equally important, particularly for older participants with potential hearing difficulties.

Given the length and complexity of the study protocol, a visual guideline was developed summarizing the main activities of each visit in a clear format. This helped participants and caregivers understand what to expect at each stage, reducing anxiety and improving adherence.

## 6 Discussion

**Figure 2:**
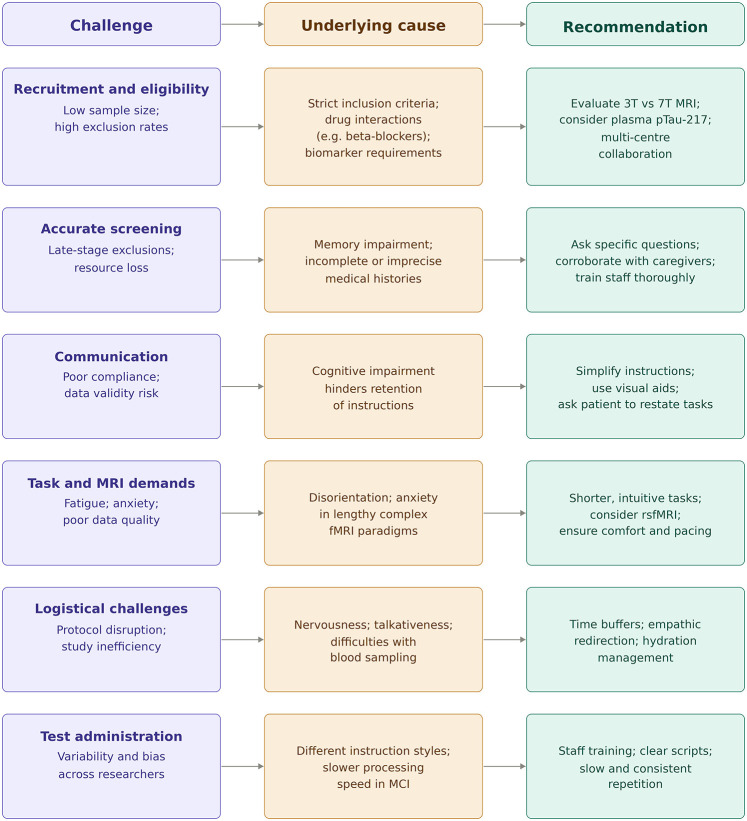
Shows the main challenges of a pharmacological 7T MRI study with AD population, underlying causes and recommendations. This is an ongoing study.

As the prevalence of Alzheimer's disease continues to rise globally, pharmacological MRI studies are central to understanding early disease mechanisms and evaluating potential interventions. Yet, the methodological and logistical challenges of conducting such studies remain largely undocumented. We argue that openly reporting these challenges, and the practical solutions developed in response, is essential for advancing the field and improving the feasibility of future research (Figure 2).

The challenges we encountered primarily arise from the interaction between stringent study designs and the cognitive and clinical characteristics of the target population. Framing these issues in terms of their underlying causes enables the formulation of practical and transferable recommendations.

### 6.1 Participant recruitment and eligibility

A central challenge concerns participant recruitment and eligibility. Strict inclusion criteria and complex study protocols substantially constrain the eligible sample: in our atomoxetine study, pharmacological requirements alone reduced it by 27.5%, largely due to drug interactions with commonly prescribed medications such as beta-blockers. Requirements for biomarker confirmation or ultra-high-field imaging further increase selectivity, and 62 out of 479 screened participants (13%) were ultimately not eligible for 7T MRI, mainly because of metallic implants (*n* = 32), missing surgical documentation (*n* = 12), tinnitus (*n* = 6), and claustrophobia (*n* = 5). The root cause lies in the fact that not all exclusion criteria carry equal weight: some are safety-driven, others are design-driven, and conflating the two leads to unnecessary sample loss. Ferromagnetic implants remain an absolute contraindication, but growing evidence on 7T-compatible dental retainer devices suggests that automatic exclusion may be overly conservative in some cases. Similarly, excluding participants solely due to missing surgical reports – rather than confirmed incompatible material – may unnecessarily reduce sample size where clinical judgment could guide the decision. To address this, we recommend that researchers critically evaluate each exclusion criterion and distinguish those that are truly non-negotiable from those that could be relaxed without compromising safety or scientific validity. Where high spatial resolution is not required, 3T MRI offers a cheaper, more accessible alternative that would circumvent 7T-specific incompatibilities entirely. For biomarker confirmation, plasma phospho-tau 217 is a promising non-invasive alternative to lumbar puncture, with recent evidence suggesting excellent diagnostic accuracy. Expanding recruitment through collaborations with multiple clinical centers using harmonized diagnostic criteria can further increase sample size.

### 6.2 Screening and communication

Closely related is the challenge of accurate screening and eligibility assessment. Memory impairments inherent to Alzheimer's disease make it difficult for participants to accurately report their medical history, increasing the risk of missed exclusion criteria during screening. In our study, however, only 12 out of 476 contacted participants were excluded during medical screening and six during experimental testing – an outcome we attribute to thorough telephonic pre-screening and well-trained research assistants. We recommend asking specific, targeted questions rather than general ones, and systematically verifying information with caregivers or clinicians when needed. Cognitive impairment also affects participants' ability to understand study procedures and retain instructions, directly impacting data validity. The underlying cause is a mismatch between standard research communication practices and the cognitive profile of this population, where reduced processing speed, working memory limitations, and attention difficulties are common. Instructions should therefore be simplified, repeated, and supported with visual aids such as a card summarizing visits and procedures. Verifying comprehension by asking participants to restate instructions in their own words is a simple but effective strategy. The tone and attitude of researchers matters equally: a confident, friendly, and calm approach improves participant comfort and performance, and clear pronunciation is particularly important for older participants with potential hearing difficulties.

### 6.3 Task and MRI demands

Task-related and MRI-specific demands represent another critical barrier. The length and complexity of pharmacological MRI protocols place substantial cognitive and physical demands on participants, who may experience fatigue, disorientation, or anxiety – particularly during complex paradigms such as task-based fMRI. To reduce this burden, we recommend designing shorter, more intuitive tasks with reduced cognitive load. Resting-state fMRI or structural imaging may be more appropriate for patients with more advanced presentations. Logistical disruptions – nervousness, talkativeness, or difficulties with blood sampling – can further strain tightly scheduled protocols; an empathic but structured approach, with sufficient time buffers and attention to basic needs such as hydration, is essential.

### 6.4 Test administration

Finally, standardization of test administration remains a critical yet underestimated challenge. Variability in instruction delivery across staff members or sessions can introduce systematic bias into the data, and this risk is heightened in populations where processing speed is reduced and responses to subtle communicative differences may be amplified. Comprehensive staff training, detailed protocol documentation, and clear instruction scripts are indispensable tools to address this. When repetition is needed, information should be delivered slowly and consistently.

In conclusion, the field of pharmacological MRI research in MCI-AD and Alzheimer's disease would benefit from greater transparency about behind-the-scenes challenges. With aging populations growing worldwide and clinical studies increasing, developing shared, evidence-based protocols for this population is urgent. We hope the lessons reported here serve as a practical resource for researchers navigating similar challenges, and encourage others to document and share their own methodological experiences.

## Data Availability

The raw data supporting the conclusions of this article will be made available by the authors, without undue reservation.
