# Supplementary material for: Behind the scenes of a 7T MRI clinical study in Alzheimer's disease: challenges and recommendations for future research
Source: Front Aging Neurosci. 2026 Jun 19;18:1731961. doi: 10.3389/fnagi.2026.1731961 (PMC13328257; doi:10.3389/fnagi.2026.1731961)
Supplement: Supplementary file 1 [file Data_Sheet_1.pdf]

Supplementary Material

1 SUPPLEMENTARY DESCRIPTIONS

1.1 Participants' diagnosis

Participants were classified according to the International Working Group-2 (IWG-2) criteria for Alzheimer's disease Dubois et al. (2014). Participants with Mild Cognitive Impairment with AD biomarkers (MCI-AD) showed objective episodic memory impairment with preserved independence in activities of daily living, along with evidence of Alzheimer's pathophysiology based on abnormal cerebrospinal fluid (CSF) amyloid biomarkers (A+; typically, Aβ42 $<$ 600, suggestive of AD). A subset of MCI-AD participants also exhibited abnormal tau and/or neurodegeneration biomarkers (A+T+N+). Alzheimer's disease dementia (ADD) was diagnosed when cognitive impairment (i.e., episodic memory and at least an additional function altered) was accompanied by functional decline affecting everyday activities and supported by positive CSF biomarkers: decreased A42 levels ($<$ 60 pg/mL) together with elevated tau concentrations $>$ 60 pg/mL and evidence of neurodegeneration indicated by increased total tau and MRI-detected ventricular enlargement and hippocampal atrophy (A+T+N+).

1.2 MRI exclusion criteria

1.2.0.1 Typical 3T exclusion criteria

- Pacemaker or defibrillator systems
- Stent surgery
- Bypass surgery (if sternum closed with wire cerclage)
- Nerve or bone growth stimulator
- Large tattoos on the head, neck, and genital area (see explanation below)
- Large tattoos on the head, neck, and genital area (see explanation below)
- Inner ear implants
- Infusion pumps
- Cerebral and ocular vascular clips or metal splinters
- Claustrophobia
- Implants that are not MRI-compatible
- Non-removable piercings
- Tattoos and permanent makeup
- Tinnitus diagnosed. If the tinnitus is not diagnosed but the patient reports ear noises, we inform that ear noises may intensify during and after the scan.

1.2.0.2 Typical 7T exclusion criteria

Typical 7T MRI exclusion criteria include the criteria above specified for 3T MRI and these additional criteria:

- artificial anus
- microblading

- gunshot injury

Note. We cannot always rely on the statements made by the test subjects that no metallic material was used/inserted during a surgical procedure, and there might be a risk if the participants do not provide the surgery report. In 7T all the surgical reports are needed. In 3T there are cases where a surgical report is still required, e.g., for emergency surgery involving an abdominal incision or spinal surgery. The same applies to unspecified ear surgery.

1.3 Stop signal task

One of the tasks included in the fMRI paradigm is the well-known Stop Signal Task (SST). Originally introduced by Lappin and Eriksen (1966) and further developed by Logan and Cowan (1984)in 1984, the SST has been widely used to investigate reaction times and executive functions, particularly inhibitory control. In this task, participants are presented with various stimuli to which they must respond, inhibit their response, or withdraw an already initiated response (see Figure S1 below). The specific nature of the stimuli is especially important for participants with Mild Cognitive Impairment (MCI) or Alzheimer's Disease (AD), as storifying the task will make it easier for individuals with cognitive impairment to understand the task % of trials. Participants adopting strategies, such as deliberately delaying responses to increase their chances of success, can shift activated neural mechanisms from inhibitory control to one of decision-making. It is therefore critical to perform a ”practice SST” before the scan session. This ensures that participants understand the instructions. Therefore, it is crucial to provide clear and appropriate instructions, emphasizing that the goal is not to perform “perfectly.” This is particularly important for older adults and those with MCI, who may feel compelled to compensate for their cognitive difficulties by trying to achieve overly high performance.

2 SUPPLEMENTARY TABLES AND FIGURES

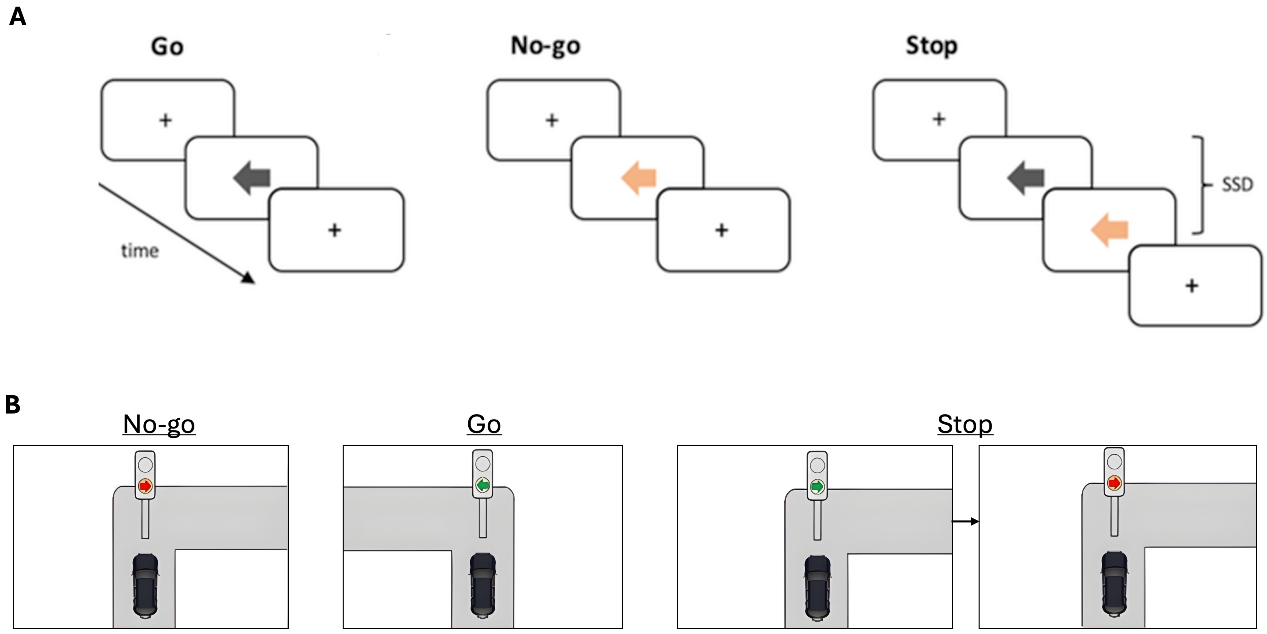

Figure S1. Figure 1 shows two different stimulus types of a stop signal task (SST): (A), the 'original' SST task designed for healthy elderly is shown. Participants perform an action when the arrow is grey (left panel), are instructed not to perform this action when the arrow is yellow, and withhold an action when the arrow turns from grey to yellow (left). (B) This task was adapted to MCI by simulating a traffic light situation: Subjects presented cars in front of traffic lights with variable colors and directions. Subjects were instructed to withhold a button press if the traffic light is red, press a button in the direction of the arrow if it is green and stop their initiated press when the traffic light turns from red to green. Image B is an adaptation of the design by Leiman and Hammerer (2022)

REFERENCES

Dubois, B., Feldman, H. H., Jacova, C., Hampel, H., Molinuevo, J. L., Blennow, K., et al. (2014). Advancing research diagnostic criteria for Alzheimer's disease: the IWG-2 criteria. *Lancet Neurology* 13, 614–629. doi:10.1016/S1474-4422(14)70090-0

Lappin, J. S. and Eriksen, C. W. (1966). Use of a delayed signal to stop a visual reaction-time response. *Journal of Experimental Psychology* 72, 805–811

Leiman, M. and Hammerer, D. (2022). An atomoxetine intervention in healthy older adults: Study Protocol. In *SFB International Symposium* (Germany)

Logan, G. D. and Cowan, W. B. (1984). On the ability to inhibit thought and action: A theory of an act of control. *Psychological Review* 91, 295–327
